# Bioinspired biliverdin/silk fibroin hydrogel for antiglioma photothermal therapy and wound healing

**DOI:** 10.7150/thno.47682

**Published:** 2020-09-23

**Authors:** Qing Yao, Qing-Hua Lan, Xinyu Jiang, Chu-Chu Du, Yuan-Yuan Zhai, Xiaohan Shen, He-Lin Xu, Jian Xiao, Longfa Kou, Ying-Zheng Zhao

**Affiliations:** 1 Department of Ultrasonography, The First Affiliated Hospital of Wenzhou Medical University, Wenzhou, Zhejiang, China; 2 School of Pharmaceutical Sciences, Wenzhou Medical University, Wenzhou 325035, China; 3 Department of Pharmacy, The Second Affiliated Hospital and Yuying Children's Hospital of Wenzhou Medical University, Wenzhou 325035, China; 4 Department of Oncology, Affiliated Cixi Hospital, Wenzhou Medical University, Cixi, 315300, China.

**Keywords:** biliverdin, silk fibroin, hydrogel, photothermal therapy, wound healing

## Abstract

**Rationale:** Photothermal therapy employs the photoabsorbers to generate heat under the near-infrared (NIR) irradiation for thermal tumor ablation. However, NIR irradiation might damage the adjacent tissue due to the leakage of the photoabsorbers and the residual materials after treatment might hinder the local healing process. A bifunctional hydrogel that holds both photothermal property and potent pro-healing ability provides a viable option to resolve this issue.

**Methods:** In this study, we developed a bioinspired green hydrogel (BVSF) with the integration of bioproduct biliverdin into natural derived silk fibroin matrix for antiglioma photothermal therapy and wound healing.

**Results:** The BVSF hydrogel possessed excellent and controllable photothermal activity under NIR irradiation and resulted in effective tumor ablation both in vitro and in vivo. Additionally, the BVSF hydrogel exerted anti-inflammatory effects both in vitro and in vivo, and stimulated angiogenesis and wound healing in a full-thickness defect rat model.

**Conclusion:** Overall, this proof-of-concept study was aimed to determine the feasibility and reliability of using an all-natural green formulation for photothermal therapy and post-treatment care.

## Introduction

As the most malignant primary central nervous system tumor, glioblastoma has high morbidity, mortality, and recurrence rate 1. Resection operation is often utilized in clinic to remove the bulk of it. However, due to the infiltrative and invasive properties, it is difficult to completely remove the malignant cells by surgery. Post-operation adjunctive treatment is necessary to avoid recurrence. Currently, radiotherapy and chemotherapy were usually used to kill the residual glioma cells. But the treatment outcome is dissatisfactory because of the severe side effects 2, 3. Phototherapy has attracted emerging attention due to its distinct advantages, especially the light-triggered and noninvasive features. Photothermal therapy (PTT) is a promising therapeutic strategy for cancer treatment, which uses photoabsorbers to generate heat to ablate cancer cells upon near-infrared (NIR) laser irradiation 4-7. Numerous photothermal materials, e.g., inorganic nanomaterials, chlorins, and porphyrins, have been investigated and displayed impressive therapeutic outcomes 8-10.

With the widening application of PTT in the treatment of multiple malignant tumors, including glioma 11, the potential off-target effect and toxic effects of photosensitizers raised health concerns. One issue for PTT is the possible leakage of photosensitizers into the neighboring health tissues and caused thermal burn injury during the irradiation treatment. The selective delivery and accumulation of theranostics in the tumor site could signigicantly improve the therapetic efficacy, diagnostic accuracy and patient safety 12-14. Additionally, repeated intracranial injections of photosensitizer was highly risky for potential infections, which could be avoided by one dose injection and repeated NIR irradiation treatments. Therefore, it is of clinic importance to confine the photothermal therapeutics at the cancerous site. In addition, the large wound after surgical resection is difficult to heal by itself. A functional hydrogel holding drug retention and pro-healing ability could offer a viable option to solve these issues for PTT. It has been reported that the injectable hydrogel could not only act as a minimally invasive formulation to deliver the photothermal agents in a semi-solid form which significantly reduced the side effects on the surrounding tissues, but also displayed significant advantages to facilitate wound healing, including filling irregular defects, providing a moist wound environment, inhibiting microorganisms, and delivering therapeutic drugs 15-19. Silk fibroin is a Bombyx mori-extracted natural structural protein biomaterial and displays excellent biocompatibility, biodegradability, antimicrobial activity, low immunogenicity, and regulation of multiple pro-healing factors 20-22. A series of silk fibroin hydrogels have been developed for various aspects of medical application, including drug delivery 23-25, wound healing 21, and tissue regeneration 26-29, and exhibited a desirable therapeutic outcome. Herein, silk fibroin was applied in our developed hydrogel for both drug delivery and wound healing.

Another problem for PTT is how to deal with the applied NIR absorbing therapeutics after the treatment. Many NIR absorbing agents and biomaterials employed in PTT are usually non-biodegradable in vivo or contain a high concentration of metal ions, which potentially exerts toxic effects on healthy cells upon administration and hinders the following healing tissue process 30, 31. Long-term retention of these photothermal materials in the body also leads to an increased risk of toxicity. It is difficult to entirely remove the photothermal therapeutics after treatment, especially for brain tumors. A secondary surgical procedure that simply aims to remove the residual photothermal formulation does more harm than good. Therefore, biodegradable and biosafe photothermal materials are urgently required. Biliverdin is a dark-green bile pigment, which is also a by-product of hemoglobin breakdown. Recently, biliverdin, the endogenous metabolite, was proven to possess high photothermal conversion efficiency, broader NIR absorption, and highly improved photostability. For instance, Xing et al. reported that biliverdin nanoagent could effectively elevate tumor temperature under mild NIR irradiation, and consequently induce efficient photothermal tumor ablation 4. In addition, biliverdin also showed a cytoprotective effect by the antioxidative and anti-inflammatory properties 32-34, which would be beneficial for wound healing. Based on these facts, biliverdin holds the potential to be utilized as not only a biodegradable photothermal agent for PTT but also a bioactive drug for the following wound healing, rather than PTT waste. What should be mentioned is that bilirubin, a bioconvertible product of biliverdin 35, which also shows anti-inflammatory, anti-cancer and photoabsorbing properties 36. However, one important distinction between bilirubin and biliverdin is that while biliverdin has a strong absorbance in the NIR region, bilirubin does not 37. In addition, the hydrophilic biliverdin is better for formulation than the hydrophobic bilirubin.

**Scheme 1:**
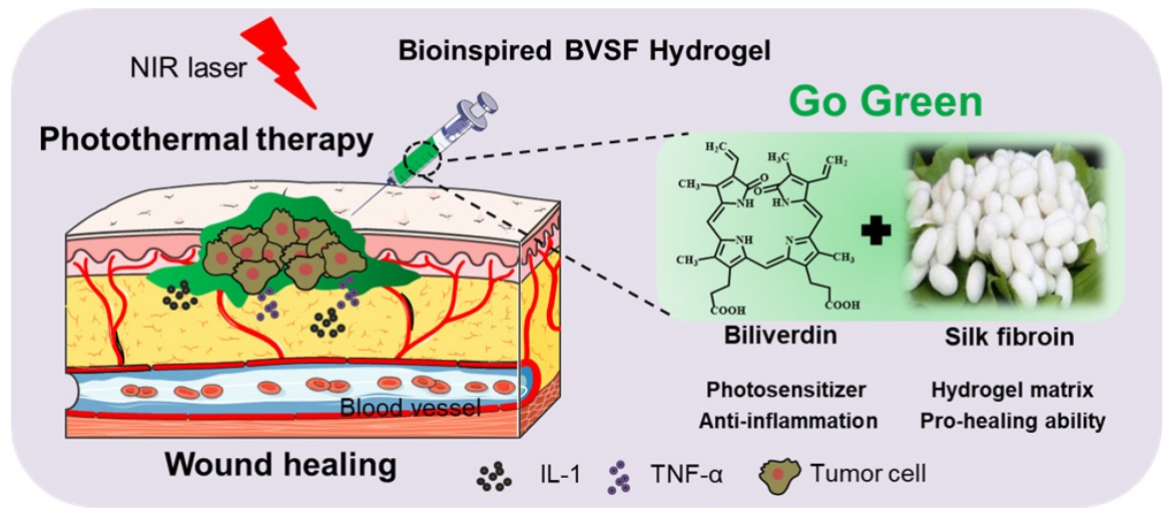
The design and fabrication of bioinspired biliverdin/silk fibroin (BVSF) hydrogel for photothermal antitumor therapy and following wound repair and regeneration.

In the human body, aged red blood cells are captured by reticuloendothelial system. Followed phagocytosed by macrophages, the hemoglobin is dissociated into globin and heme, and heme is then degraded by heme oxygenase to iron, carbon monoxide, and biliverdin. Inspired by this fact, we used a protein-based biomaterial, silk fibroin, to formulate biliverdin to develop a bioinspired green hydrogel (BVSF) (Scheme 1). This all-natural constructed and biomimetic BVSF hydrogel could not only achieve photothermal mediated ablation of glioma cells but also facilitate the wound healing process as well as tissue reconstruction. Besides the prolonged retention, the hydrogel system could also protect biliverdin from the enzymes-mediated metabolism and extent its lifetime. Herein, we described a detailed investigation of the BVSF hydrogel-mediated NIR-stimulated photothermal activity and pro-healing ability both in vitro and in vivo.

## Materials and Methods

### Materials

Bombyx mori Cocoons were purchased from Huzhou Xintiansi Biotech Co., Ltd (Huzhou, China). Biliverdin (Catalog No. 50-981-690) was bought from Thermo Fisher Scientific (Waltham, MA, US). Lithium bromide, Na_2_CO_3_, and fluorescein isothiocyanate (FITC) were from Sigma-Aldrich (St Louis, MO). Indocyanine green (ICG) (Catalog No. MB4594) was obtained from Meilunbio VR (Dalian, China). Fetal bovine serum (FBS), trypsin, phosphate buffered saline (PBS) and Dulbecco's Modified Eagle's Medium (DMEM) were purchased from Life Technologies (Grand Island, NY). The cell lines, including C6, U87, GB261 and RAW 264.7, were obtained from the Institute of Biochemistry and Cell Biology, Shanghai Institutes for Biological Sciences, Chinese Academy of Sciences (Shanghai, China). The antibodies of Anti-Ki67 (ab15580,), anti-α-SMA (ab52575), and anti-IL-1 (ab9722) were obtained from Abcam (Abcam, Cambridge, MA, US). Anti-TNF-α (52b82) antibody was bought from Santa Cruz Biotechnology (Dallas, TX, US).

### Preparation and characterization of BVSF hydrogel

The silk fibroin was extracted from Bombyx mori Cocoons as our previously reported method 38. Silk fibroin was dissolved in distilled water at the concentration of 2% (wt./v). Biliverdin solution was obtained by dissolving in distilled water at the concentration of 100, 200, 300, 400, and 500 µg/mL. The BVSF hydrogel was prepared by two steps. Firstly, the biliverdin solution was added into the silk fibroin solution drop by drop with constant stirring to obtain a pre-BVSF solution. Before use, the mixed solution was gelatinized by ultrasound at 100 W for 4 min. The appearance of prepared BVSF hydrogel was photographed by a camera. ICG loaded silk fibroin hydrogel (ICGSF) was prepared by a same method using ICG instead of biliverdin. When FITC was used to label the BVSF, a proper amount of FITC dissolved in ethanol was added into the prepared pre-BVSF solution with constant stirring for 1 h in the dark. The mixed ethanol in the solution was removed by the reduced pressure to obtain FITC-labeled pre-BVSF, which was further gelatinized by ultrasound at 100 W for 4 min to make FITC-labeled BVSF hydrogel.

The microstructure of BVSF hydrogel before and after gelation was visualized by scanning electron microscope (SEM; Hitachi, H-7500, Japan) technique. The BVSF hydrogels were firstly frozen and lyophilized with a vacuum freeze-dryer for 24 h. The dehydrated specimens were crosscut, and the surface was sputtered for SEM visualization. The dehydrated BVSF hydrogel was further used for Fourier Transform Infrared Spectroscopy (FTIR; Thermo Fisher Nicolet FTIR 5700 spectrometer, Thermo Scientific, FL, USA) analysis by the KBr disk method. For each measurement, 24 scans were co-added with a resolution of 4 cm^-1^ and the wave number ranging from 1000 to 4000 cm^-1^. To investigate the characteristics of BVSF, Biliverdin, silk fibroin, BVSF were diluted in pure water, and the ultraviolet-visible (UV-Vis) spectrum from 300 to 1000 nm was recorded (TU-1901, Beijing). The same sample was used for fluorescence spectra (RF-6000, Shimadzu, Japan). The excitation length was 465 nm, and the recorded spectra was ranging from 500 to 900 nm.

BVSF hydrogels were prepared as described above, at the different solid concentrations (1%, 2 %, 3%) in distilled water. The rheological properties of the hydrogels were tested by using a rotary rheometer (DHR-2, TA, USA). The viscosity of hydrogels was measured under flow step as a function of shear rate from 0.1 s^-1^ to 90 s^-1^ to study the shear thinning behavior of the hydrogel. Time sweep measurements were performed at fix strain (γ = 0.1%), frequency (ω =15 rad/s) and temperature (T = 37 °C). Frequency sweep measurements were performed from 0.1 to 100 rad/s at fix strain (γ = 0.1%) and temperature (T = 37 °C). The frequency sweeps were measured after storage modulus reached an equilibrium state. All measurements were performed in the linear viscoelastic region.

### Photothermal properties of BVSF hydrogel

The photothermal properties of BVSF hydrogel were evaluated under NIR (808 nm) irradiation. Briefly, the BVSF hydrogel (1 mL) with various biliverdin concentration was placed in a 2 mL glass bottle, and then exposed to a NIR irradiation source (FU808ADX-F34; China) at three different power intensity. The temperature changes were monitored using an infrared (IR) thermal camera (FLIR ONE PRO; China). The in vivo photothermal properties of BVSF hydrogel were also evaluated by recording the tumor surface temperature and the thermal photograph of tumor-bearing mice using an IR camera while receiving NIR irradiation.

The photothermal conversion efficiency (*η*) of BVSF was calculated according to the reported method 4, 39. A standard formula was used as follows:


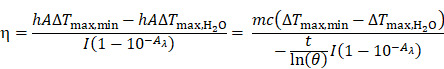


“*m*” represents the quality of BVSF. “*c*” is the specific heat capacity of water. “
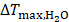
” is the temperature change of water at the maximum steady-state temperature. “
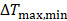
” is the temperature change of BVSF at the maximum steady-state temperature. “*t*” is the corresponding time. “*θ*” is the dimensionless driving force. “*I*” represents the power of the laser. “*A_λ_*” is the absorbance at 808 nm.

### Fluorescence-based live/dead staining assay

Fluorescence-based live/dead assays were carried out as previously reported 40 with modification. Briefly, C6 or GL261 cells were seeded into 6-well plates and incubated with or without BVSF for 24 h. Due to the gel state could affect the detection of cell viability and the photothermal conversion ability of BVSF in solution state is consistent with that in hydrogel state, we thereby used liquid pre-BVSF solutions here for live/dead staining assay. The cells were then washed before 8 min NIR laser treatment and then stained with calcein acetoxymethyl/ethidium homodimer-1 (calcein-AM/EthD-1) for 30 min. Cells were washed and then visualized with an Inverted fluorescence imaging system (ECLIPSE TI-S, NIKON, Japan). The cell viability rate was calculated by the ratio of total live/total cells (live and dead).

### In vitro cytotoxicity assay

Cytotoxicity was evaluated using 3-(4,5-dimethylthiazol-2-yl)-2,5-diphenyltetrazolium bromide (MTT) assays as we previously reported 41, 42. Briefly, cells were seeded into 96-well plates for 12 h. Then, culture media were replaced with fresh media containing various drug treatments. Then cells were irradiated with a NIR laser at 2 W/cm^2^ powers for 3 min. After 24 h, cells were washed and further incubated in fresh media containing 0.5 mg/mL MTT solution (100 µL). After 4 h, the medium was replaced with dimethyl sulfoxide (DMSO) (150 µL) in each well to dissolve the resulting formazan crystals. After briefly shaking, absorbance was measured at a wavelength of 540 nm using a microplate reader (MultiskanMK3, Thermo, Waltham, MA). Due to the same reasons described in live/dead assay, we also used liquid pre-BVSF solutions here for MTT assay.

### Determination of proinflammatory cytokines

ELISA kits (Multi Sciences, Hangzhou, China) were used for the determination of the proinflammatory cytokines, TNF-α and IL-1, in the lipopolysaccharide (LPS)-stimulated RAW 264.7. Briefly, the RAW 264.7 cells were first stimulated with LPS for 6 h, and then treated with different drug treatments for 24 h. Then, the medium supernatant was collected from RAW 264.7 cells and analyzed following the manufacture's protocol.

### In vivo retention time of BVSF hydrogel

All animal experiments were performed under the approval and guidance of the Institutional Animal Care and Use Committee of Wenzhou Medical University. The subcutaneously implanted tumor model was developed as previously reported 41, 43, 44. Briefly, C6 cells (1 × 10^7^) were injected into the right flank of male BALB/c nude mice to establish the subcutaneous glioma tumor models. Tumor volume was calculated as (tumor length) × (tumor width)^2^/2. When the tumor volume reached approximately 100~200 mm^3^, C6 tumor-bearing mice were randomly divided into two groups (n=3). The two groups were receiving intratumorally injection of 200 µL FITC labeled BVSF hydrogel with or without ultrasonication process; in other words, pre-BVSF solution and BVSF hydrogel. The biliverdin concentration was fixed at 0.2 mg/mL. The fluorescence images were monitored at the determined time (2 h, 6 h, 12 h, and 24 h) by a whole-body imaging system equipped with an excitation filter (488 nm) and emission filter (525 nm) was used to collect fluorescence signals. After 24 h post-injection, the mice were sacrificed, and the tumor was collected for biliverdin concentration analysis. For drug determination, 0.25 g tumor tissue was homogenized in 0.5 mL PBS for 1.5 min, and then 1.0 mL chloroform was added, and the sample was vortexed for 3 min to extract the biliverdin. After that, the sample was centrifuged in 3000 rpm for 3 min to collect the lower chloroform-containing organic phase. Then chloroform was evaporated under a nitrogen stream, and the residue was dissolved in DMSO for UV-vis analysis. This method has been validated, and the linear range, recovery, and matrix effect are all acceptable. The amount of biliverdin in test group minus that in the control group (without any treatment) was presented in the results.

### In vivo antitumor therapy

When the tumor diameter reached 100~200 mm^3^, the C6 tumor-bearing BALB/c nude mice were randomized into four groups (n = 5): Saline (control), 2% SF hydrogel, ICGSF (80 µg/mL), and BVSF hydrogel (200 µg/mL). All mice were treated with 808 nm irradiation (2 W/cm^2^, 10 min) on day 1, day 4, day 7, and day 10. The body weight and tumor volume of mice were measured every day during the whole experiment period. On day 14, the mice were sacrificed, and the tumor tissues were collected, weighed, and photographed. The tumor tissues were also fixed, embedded in paraffin sectioned into 5-μm-thick slices. The tumor samples were stained with hematoxylin-eosin (H&E), Terminal deoxynucleotidyl transferase dUTP nick end labeling (TUNEL), Ki67, as previously reported 41. Other major organs (heart, liver, spleen, lung, and kidney) were also collected and paraffined for H&E staining.

### In vivo wound healing

A full-thickness skin defect rat model was developed as previously reported 45. Briefly, a circular full-thickness skin wound (diameter, 10 mm) was made on the back of SD male rats. The wounds were treated with three different treatments, PBS (control), silk fibroin hydrogel (SF), and BVSF hydrogel (BVSF). Then the wounds were further covered by Tegaderm after drug treatment. The wounds were photographed every 3 days, and the wound area was quantitatively analyzed via Image J software. The relative wound area was calculated as the ratio of the remaining wound area to the original wound area. On day 7, 14, and 21, the entire wound was excised, fixed, and sectioned into 5 μm-thick slices for H&E and Masson's staining. Image J with a built-in Masson Trichrome color deconvolution was used to quantify the collagen positive area referring to a reported method 46, and the total blue area was normalized to the total tissue area. The hair follicles were counted by the skin pore in three different field, and the results were presented as the skin pore number per mm^2^. Immunohistochemistry of the samples was also performed using α-SMA, TNF-α, and IL-1 antibodies (Abcam, UK), and the quantitative analysis was performed by Image J.

### Statistical analysis

All the data were expressed as mean ± standard deviation. All statistical analysis was carried out using GraphPad Prism 5.0 (GraphPad Software, San Diego, CA). Statistically significant differences (P<0.05) was obtained by the analysis of variance and student t-test.

## Results and Discussion

### Preparation and characterization of BVSF hydrogel

PTT with high efficiency and minimal invasiveness is a promising approach for cancer treatment, but the photothermal absorbing materials have been stifled by safety concerns, including the potential toxicity of materials and retention of the materials might hinder the subsequent wound healing. In order to address the safety concerns and achieve repeated PTT, we prepared an all-natural derived green BVSF hydrogel by integrating the bioproduct biliverdin into the silk fibroin matrix.

**Figure 1:**
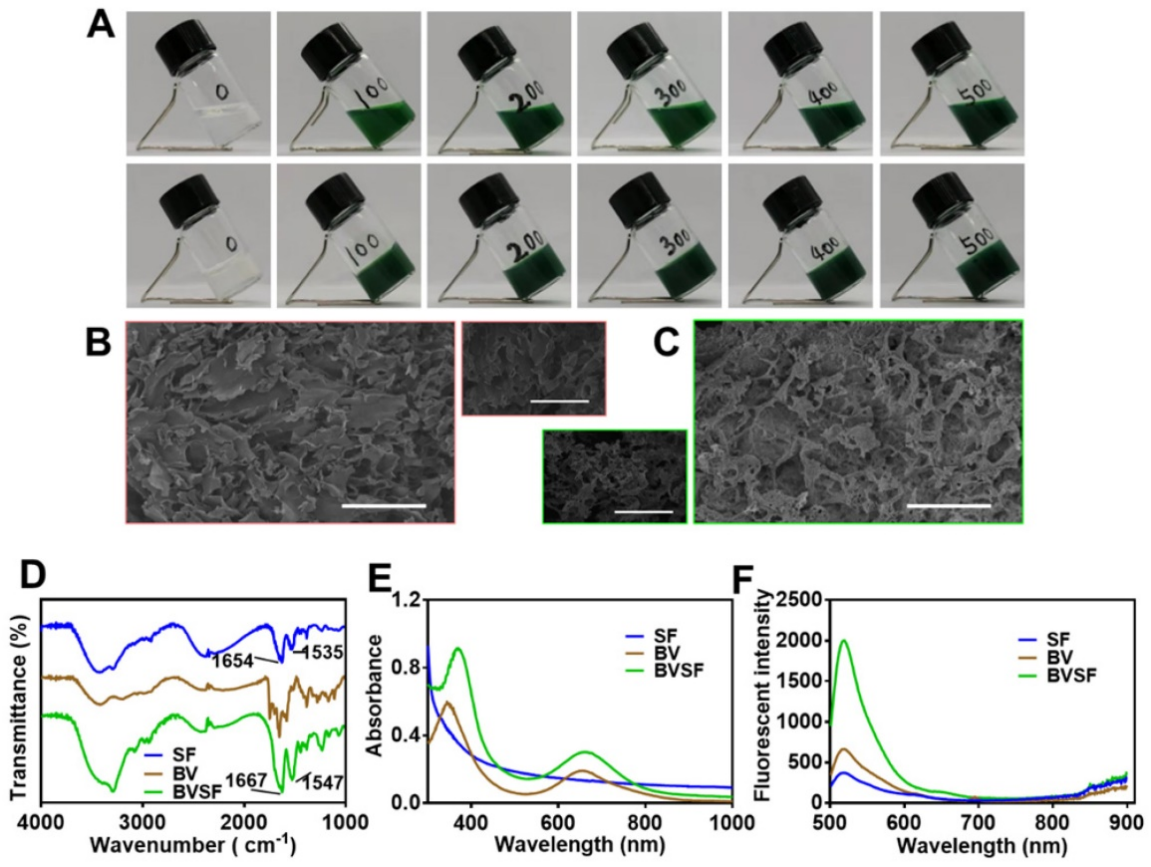
The morphological and rheological properties of biliverdin/silk fibroin (BVSF) hydrogel. A) The photographs of the preBVSF solution (the first row) and BVSF hydrogel (the second row); B) SEM images of the pre-BVSF solution (scale bar = 100 μm); C) SEM images of BVSF hydrogel (scale bar = 100 μm); D) The FT-IR spectrum, E) UV-Vis spectra, and F) fluorescent spectra of the silk fibroin (SF), biliverdin (BV), and BVSF hydrogel.

After administration, the injectable hydrogel could quickly undergo solidification and stay in the tumor site for a prolonged time, which also reduced the systemic toxicity and avoided repetitive dose. Silk fibroin solution was prepared as previously reported 47 before mixing with the biliverdin solution thoroughly. The obtained BVSF solution showed no distinct phase separation and displayed as a uniform green solution (pre-BVSF) (Figure 1A), indicating excellent compatibility between biliverdin and silk fibroin. The injectable ultrasonication-induced silk hydrogel was prepared as previously reported 48, 49. Briefly, the pre-BVSF solution was subjected to ultrasonication before loaded into a syringe and placed at 37 °C for 4 min to allow gelation and formed a hydrogel structure (Figure 1A). The quick gelation process was beneficial for the following application 50. Without ultrasonication, the pre-BVSF solution will be gelated after 6 h at 37 °C. The ultrasonication could significantly facilitate the gelation of BVSF. The injectable property of silk fibroin makes them relatively easy to operate with low invasiveness. As the concentration of biliverdin increased, the color of the obtained BVSF hydrogel became darker. The microstructure of the BVSF system exhibited a distinct change after gelation (Figure 1B-C), and BVSF hydrogel presented a clear porous network structure and high surface area. The porous structure of BVSF hydrogel could provide a favorable condition for cell migration and the growth of capillaries, promoting the healing process. In addition, the connected pores also facilitated the exchange of oxygen and nutrients, which avoided the formation of dead space in the tissue.

The rheological properties of BVSF was further investigated 51. The viscosity of BVSF dramatically decreased with the shear rate increasing from 0.1 to 90 s^-1^ (Figure S1A). Such shear-thinning ability enabled our BVSF hydrogel with distinct injectability. In addition, the viscosity of BVSF was positively correlative with the solid concentration. Time scanning was performed, and the dynamicmodulus (G', G'') of BVSF was shown in Figure S1B. The storagemodulus (G') of each hydrogel is almost one order of magnitude higher than loss modulus (G''), suggesting that BVSF hydrogels could maintain the mechanical stability at 37 °C after gel formation. The frequency sweep results of these hydrogels showed that G' was always larger than G'' (Figure S1C), which indicates the elastic behavior and a gel-like state of BVSF hydrogels. The mechanical properties of BVSF hydrogels showed a dependence on the solid concentration. Collectively, BVSF hydrogel displays excellent injectable and mechanical properties, which were in favor of clinical applications.

Silk fibroin is a biocompatible protein with excellent drug loading properties and suitable characteristics for the pharmaceutical process. The BVSF hydrogel was analyzed by FTIR (Figure 1D) to evaluate the typical absorption bands of the silk fibroin. The infrared spectrum of silk fibroin showed three random crimp peaks at 1654 cm^-1^ (amide I),1535 cm^-1^ (amide II) and 1235 cm^-1,^ (amide III), indicating the coexist of α-helix and β-sheet structures. However, the amide I, amide II and amide III bands shifted to 1664 cm^-1^, 1547 cm^-1^ and 1230 cm^-1^ respectively in the presence of the biliverdin. The absorption bands, especially amide I and amide II, were changed to higher wavelengths after silk fibroin blended with biliverdin 52. It was suggested that silk fibroin could interact with biliverdin which increased random coil structure. The potential hydrogen bond, as well as electronic interaction between hydroxyl of the silk fibroin and carboxyl groups of biliverdin, might contribute to the peak shift. Then, we further evaluated the absorption (Figure 1E) and the fluorescence spectrum (Figure 1F) of the BVSF system to verify the molecular interaction between silk fibroin and biliverdin. The blank silk fibroin showed decreased absorption with the increase of wavelength, and no absorption peak was observed in the wavelength range of 300-1000 nm. Free biliverdin solution showed two characteristic absorption peaks at 344 nm and 654 nm, which shifted to 370 nm and 659 nm in the presence of silk fibroin. It was speculated that the hydrogen bond or the electronic interaction between the silk fibroin and biliverdin contributed to the red-shift of absorption peaks assigned to biliverdin. The fluorescence spectrum results also verified the existence of molecular interaction between the two components of the BVSF system. As displayed in Figure 1F, the fluorescence intensity of biliverdin was significantly increased after complexation with silk fibroin, which attributes to the intermolecular interaction in the BVSF system. Therefore, silk fibroin was suitable to formulate biliverdin by an intermolecular interaction, which facilitated the formation of the BVSF hydrogel structure for biomedical application.

### Photothermal Properties of BVSF hydrogel

**Figure 2:**
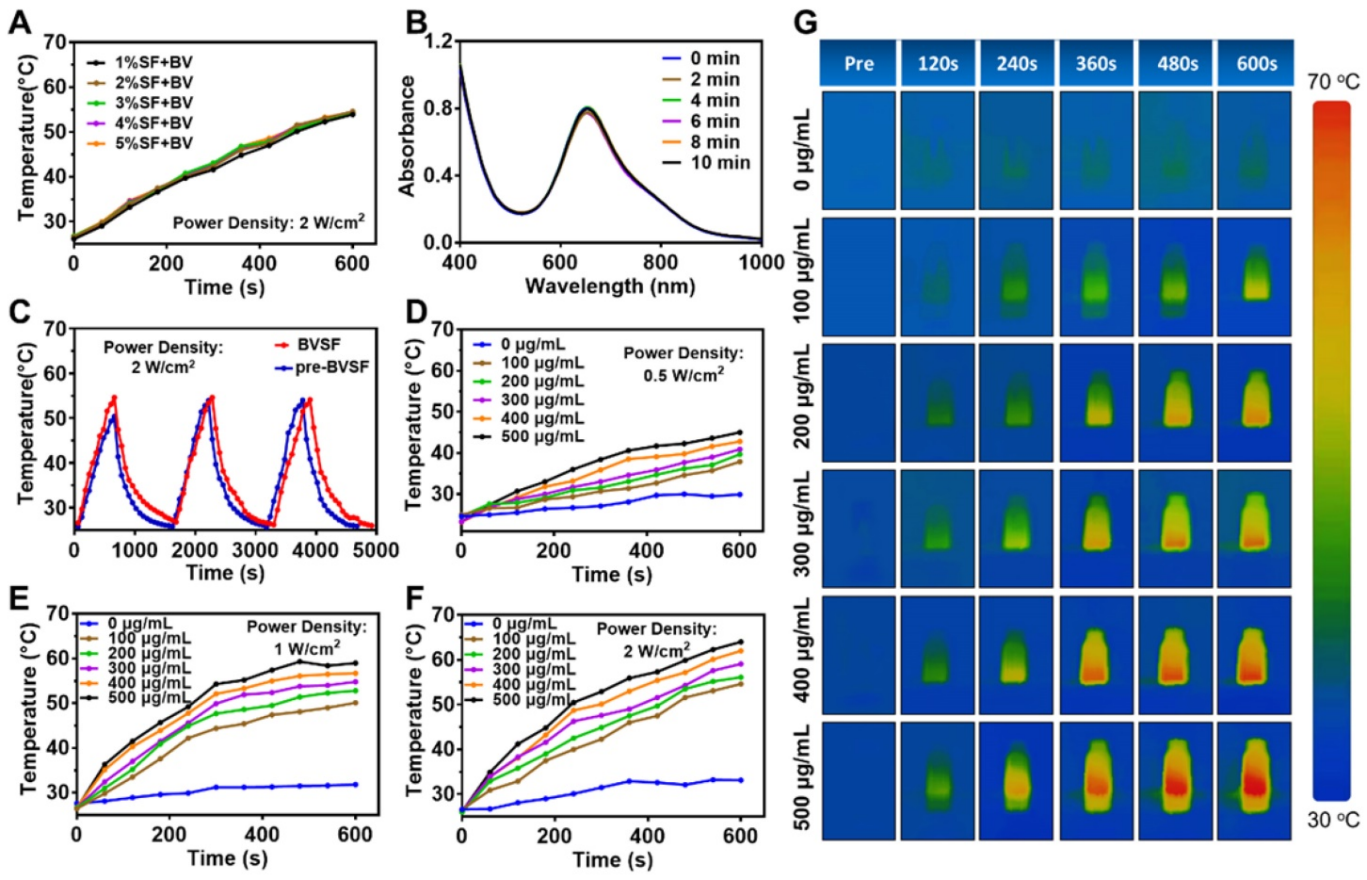
The photothermal properties of biliverdin/silk fibroin (BVSF) hydrogel. A) The photothermal heating curves of BVSF hydrogel with different silk fibroin concentration; B) The UV-vis-NIR spectrum of BVSF hydrogel (100 µg/mL); C) The photothermal stability of the pre-BVSF solution and BVSF hydrogel; The photothermal heating curves of BVSF hydrogel with different biliverdin concentration by D) 0.5 W/cm^2^ laser, E) 1 W/cm^2^ laser, and F) 2 W/cm^2^ laser, respectively. G) The photothermal images of BVSF hydrogel by 2 W/cm^2^ laser.

It has been shown that biliverdin exhibited NIR absorbing capability resulted in photoacoustic and photothermal properties 53, 54, making it potentially useful for hyperthermic cancer treatment. We investigated the photothermal properties of BVSF hydrogel, as presented in Figure S2 and Figure 2. Silk fibroin could interact with biliverdin and potentially enhance the photothermal characteristics of biliverdin. Biliverdin showed a distinct NIR absorption ability and resulted in a significant elevated aqueous temperature (Figure S2). The addition of silk fibroin into the biliverdin solution increased the efficiency of biliverdin in converting light into heat as evidenced by the higher temperature after 10 min NIR irradiation (Figure S2A). It was indicated that biliverdin contributed to the photothermal transformation, which could be magnified by the interaction with silk fibroin. A further increased concentration of silk fibroin (Figure 2A), however, showed no observed effect on the photothermal conversion ability of the BVSF system. Based on these results, the photothermal efficiency of BVSF hydrogel was calculated using a previous reported method 4, 39. The results showed that BVSF hydrogel had a photothermal conversion efficacy (*η*) of 30.28%, which was higher than that of bare biliverdin (28.94%), indicating the enhanced photothermal property. The photothermal conversion efficacy of BVSF was comparable to the reported representative organic (under 40%) and inorganic (10-20%) photothermal nanomaterials, and also to the reported biliverdin-involved nanomaterial (34.3%) which integrated Mn^2+^ 4. It should be noted here that no metal ion was employed in BVSF hydrogel, and still resulted in a high photothermal conversion efficacy. The increased photothermal conversion of BVSF might be attributed to the enhanced stability of biliverdin in a hydrogel, also termed “supramolecular photothermal effects” 55, 56. Biliverdin could be significantly affected by oxygen or reactive oxygen species (ROS) due to the weak chemical bond, which resulted in the oxidation/degradation of biliverdin 57. The hydrogel system could trap biliverdin inside a complex and make biliverdin molecules isolated, which could protect biliverdin from oxidation and decomposition, therefore maintaining its photophysical and photochemical properties. The ultraviolet-visible-near infrared (UV-vis-NIR) absorption of BVSF hydrogel was recorded (Figure 2B), which showed that BVSF had a broad and robust UV-vis-NIR light absorption with the peak reached at 668 nm (within the NIR window region, 650 ~ 950 nm). Additionally, the absorbance of BVSF had good stability even after prolonged exposure to NIR irradiation (Figure 2B). To further study the photothermal stability of BVSF hydrogel, the cyclical laser irradiation was also conducted under 808 nm at a power density of 2 W/cm^2^ (Figure 2C) in three repeated cycles of irradiation on and off. As displayed in Figure 2C, the BVSF system at both aqueous state and solid-state did not exhibit any visible decay during the observation period, suggesting the potential of BVSF hydrogel for reusable photothermal applications.

We further evaluated the temperature profiles of BVSF hydrogel system by controlling the biliverdin concentration (0, 100, 200, 300, 400, 500 μg/mL) under NIR irradiation with a power intensity of 0.5 W/cm^2^ (Figure 2D), 1 W/cm^2^(Figure 2E), and 2 W/cm^2^ (Figure 2F). The corresponding IR thermal images were recorded at 2 W/cm^2^ irradiation were also recorded (Figure 2G). As expected, the increase of biliverdin concentration resulted in a faster heating up process and a higher final temperature. At the biliverdin concentration of 500 μg/mL, the temperature of BVSF hydrogel quickly reached 64 °C after 10 min of 2 W/cm^2^ NIR irradiation (Figure 2F-G). Meanwhile, the temperature of BVSF hydrogel with 100 μg/mL was only 53 °C after the same NIR irradiation treatment. In contrast, the temperature of the silk fibroin solution almost unchanged. Similar results were also obtained at lower NIR intensity irradiation. These results indicated that biliverdin acted as the main contributor to the photothermal transformation of BVSF hydrogel, and the BVSF hydrogel possessed the biliverdin concentration-dependent photothermal performance. Additionally, the temperature profiles of BVSF hydrogel under different light intensity in Figure 2D-F and Figure S2B showed that the final temperature increased as the applied NIR irradiation power elevated. Therefore, the photothermal capability of BVSF hydrogel highly depended on both biliverdin concentration and laser power. Taken together, the all-natural derived BVSF hydrogel possessed excellent photothermal conversion properties and showed a rapid temperature elevation under NIR laser irradiation.

### In vitro BVSF hydrogel mediated photothermal ablation of cancer cells

**Figure 3:**
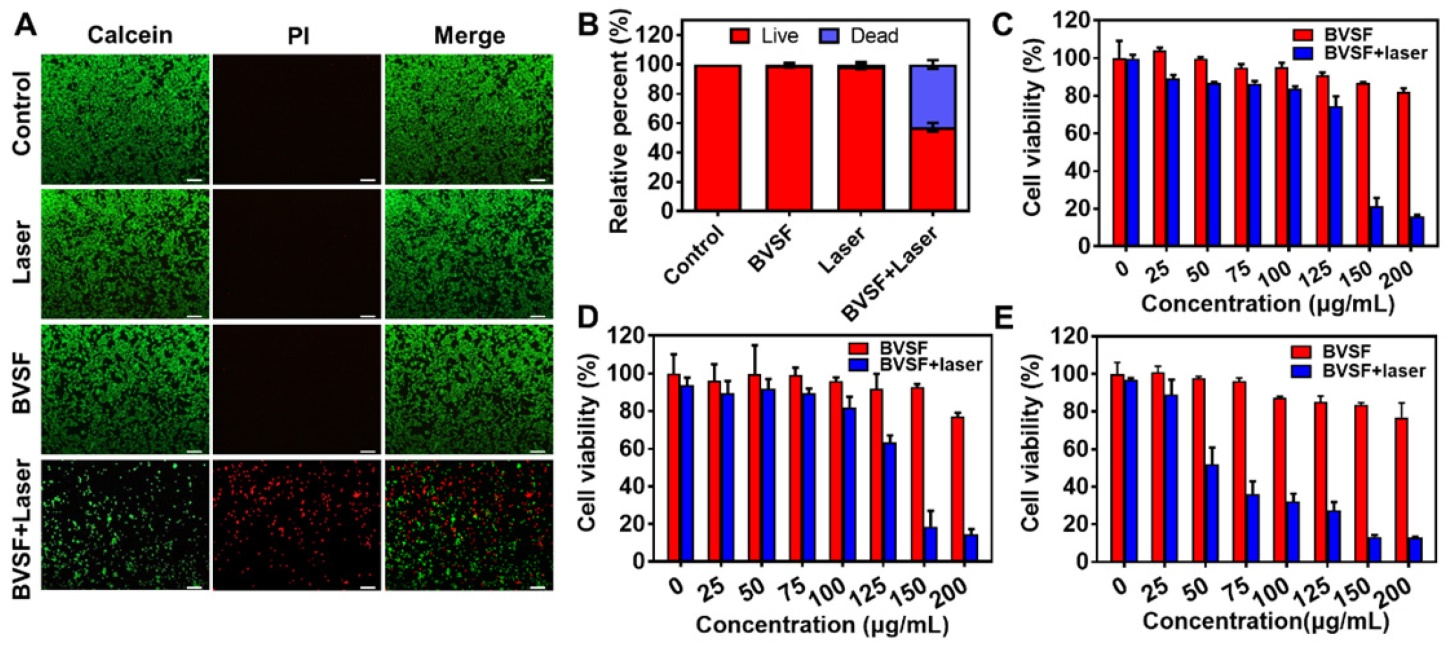
The photothermal mediated kill effect on cancer cells of biliverdin/silk fibroin (BVSF) hydrogel. A) The live/dead staining images (Green signal indicates live cells, and the red signal indicates dead cells) of C6 cancer cells after various treatments. The concentration was 200 µg/mL (calculated as biliverdin), and the laser power density is 2 W/cm^2^ (808 nm, 8 min). Scale bar = 100 µm. B) Calculated live/dead ratios of C6 cancer cells with various treatments. Relative cell viability of C) C6, D) GL261, and E) U87 cells after treatments with BVSF in the presence or absence of NIR irradiation (808 nm, 3 min).

The thermal ablation of tumor cells with PTT is a promising approach for antitumor therapy. By local administration of photosensitizers and minimally invasive NIR irradiation, hyperthermia-induced by photothermal can be controlled to minimize the damage toward non-targeted tissues. To verify the photothermal antitumor efficacy of BVSF hydrogel, we compared and investigated the cytotoxicity of BVSF hydrogel with or without NIR irradiation. Both live/dead fluorescent staining (Figure 3A-B and Figure S3) and MTT assay (Figure 3C-E) have been adopted to assess the cell apoptosis and viability. As presented in Figure 3A-B, only negligible dead cells were observed in the BVSF group that did not receive irradiation, indicating high compatibility of developed bioinspired BVSF hydrogel. Similar results were also obtained in GB261 and U87 cells (Figure S3). Compared to the laser group that exposed to the same laser intensities, BVSF treated cancer cells displayed distinct cellular death, evidenced by the much stronger red fluorescence (Figure 3A and Figure S3A-B). These results suggested that BVSF-mediated photothermal ablation was dependent on the laser irradiation. MTT results indicated that BVSF hydrogel itself did not show a significant cytotoxic effect on all tested cell lines (C6 cells, GB261 cells, and U87 cells), with over 80% cell viability at a concentration of 200 μg/mL, but displayed significant anticancer effect while exposing NIR irradiation. Although the cell response toward this cell-killing effect slightly different in each cell line, the BVSF mediated photothermal killing effects showed an obvious biliverdin concentration-dependency (Figure 3D-E). It was consistent with the photothermal heating curves of BVSF hydrogel as presented in Figure 2D-F. Additionally, time-dependent damage by BVSF mediated photothermal ablation of tumor cells was also investigated in C6 cells (Figure S4). As expected, for a given fixed laser power of 2.0 W/cm^2^, longer laser irradiation time achieved lower cell viability before reaching a complete kill. These results demonstrated that BVSF hydrogel could be used for photothermal ablation of multiple glioma cancer cell lines, and the cell-killing rate was highly related to the biliverdin concentration as well as irradiation time.

### In vivo photothermal property and prolonged retention of BVSF hydrogel

**Figure 4:**
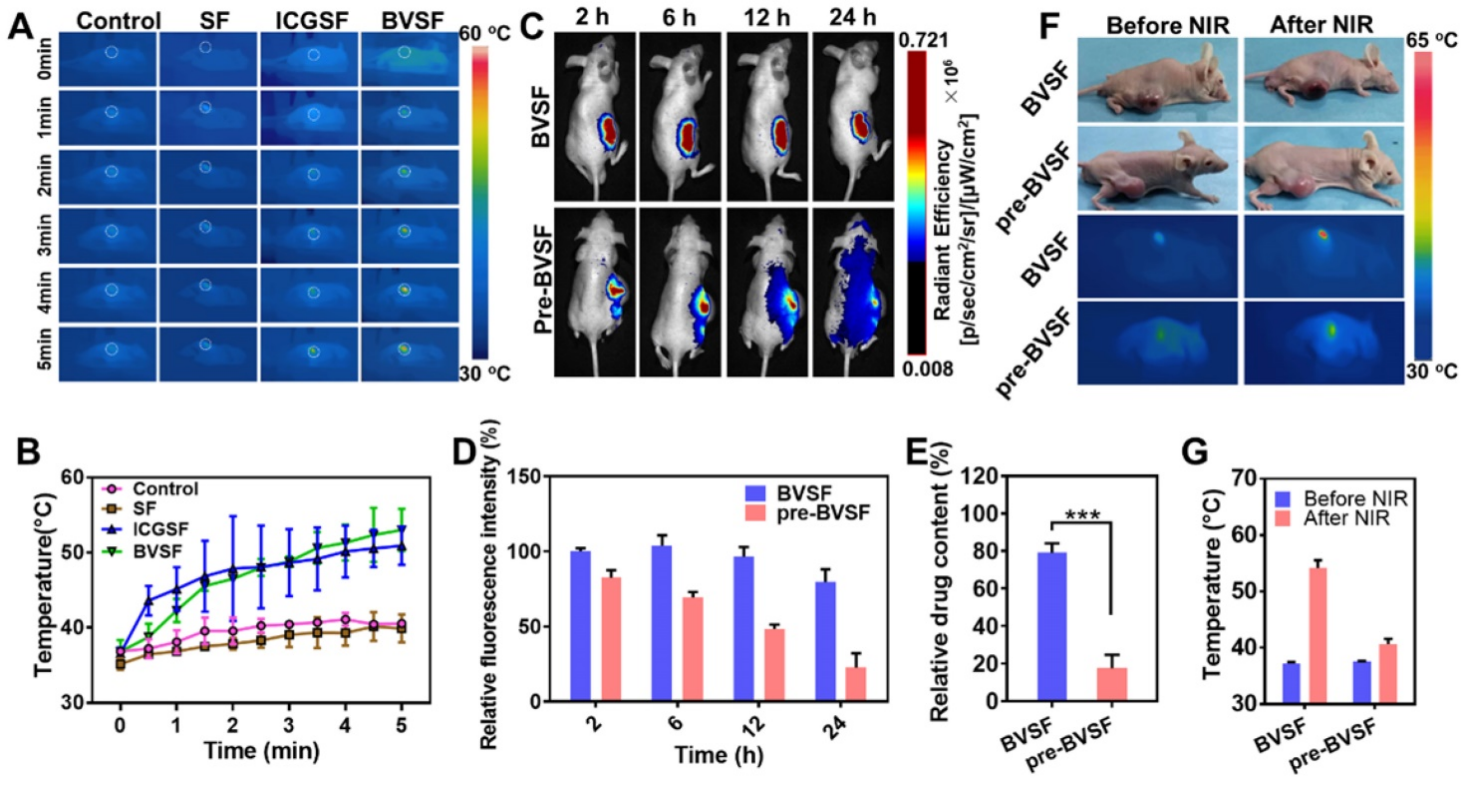
In vivo retention and photothermal activities in a tumor-bearing model. (A) The dynamic photothermal images and (B) temperature-time profile at the tumor site under NIR irradiation (808 nm, 5 min, 2 W/cm^2^). (C) The in vivo retention images and (D) semi-quantitative results of FITC-labeled BVSF hydrogel in tumor-bearing mice. (E) The remaining biliverdin at the tumor site after 24 h treatment in BVSF and pre-BVSF group. (F) IR thermal images of the BVSF hydrogel intratumorally injected mice under NIR irradiation (808 nm, 5 min) at 24 h after administration, and (F) the temperatures were recorded.

Then, in vivo NIR stimulated photothermal properties of BVSF hydrogel were investigated by irradiating the BVSF hydrogel after intratumoral injection (Figure 4A-B). The in vivo BVSF mediated photothermal property was studied in C6-bearing nude mice by monitoring the temperature change (Figure 4 A-B), with ICGSF as a control. The temperature and IR images were recorded by an IR imaging camera. The BVSF hydrogel showed a significantly elevated surface temperature, indicating that BVSF hydrogel could convert the laser energy to the thermal energy under NIR irradiation (Figure 4 A-B). The conversion capability of BVSF hydrogel was even comparable to that of the ICGSF hydrogel group. As expected, the gel area displayed a significant temperature increase compared to tissues far away from the gel (Figure 4A). Additionally, temperature profiles showed no significant difference between the silk fibroin (SF) group and the control group, which demonstrated that the biliverdin mainly contributed to the photothermal conversion in the BVSF hydrogel. This result was consistent with the in vitro temperature change of BVSF hydrogel under the NIR irradiation (Figure 2D-E).

A hydrogel system could act as a depot and keep the encapsulated theranostic agents at the pathological site, which maintained the concentration of drugs, imaging agents, and photothermal materials to reach a sufficient therapeutic level. Therefore, we investigated the in vivo retention profile of BVSF hydrogel by an in vivo imaging system (Figure 4C). The pre-BVSF solution without the ultrasonication process presented as an aqueous state was used as a control (Figure 1A). After injection, the pre-BVSF solution quickly distributed to surrounding tissues and spread to the whole body after 24 h, resulting in significantly decreased fluorescent intensity at the tumor site. On the contrary, the ultrasonication process promoted the formation of a hydrogel system, resulted in a semi-solid state of BVSF hydrogel (Figure 1A). Therefore, the formed BVSF hydrogel displayed prolonged retention in the tumor tissue compared to pre-BVSF during the whole observation period (Figure 4C). The semi-quantitative results showed that about 80% of fluorescent intensity remained in the BVSF hydrogel system (Figure 4D). These results also confirmed the successfully developed hydrogel system using biliverdin and silk fibroin after ultrasonication. We further determined the biliverdin concentration (Figure 4E) after 24 h that remained in the tumor tissues. Consistent with the imaging data, the biliverdin concentration kept maintaining at a high level while loading in a silk fibroin hydrogel matrix (Figure 4D). As indicated, about 80% biliverdin was detected in the BVSF group after 24 h, while that for pre-BVSF was not up to 20%. However, we should noted that only 20% biliverdin in BVSF hydrogel would be left in the tumor site after one weeks, which required rational design of PTT treatment time and frequency to obtain desired therapeutic outcomes. Still, as compared to the pre-BVSF group, the gel system was significantly enhance the retention of photosensitizers, and decrease the administration requirement The prolonged retention of biliverdin in BVSF group was attributed to the semi-solid structure of the hydrogel system minimizing the leakage of biliverdin. As compared to the pre-BVSF solution, BVSF hydrogel had much higher biliverdin retention in the tumor site. Even though the NIR laser could concentrate the effect in a restricted place, the retention of photosensitizers in tumor site is also essential. On the one hand, the prolonged retention time of the gel could maximize the therapeutic effect by maintaining the photosensitizer concentration at a high level during the NIR irradiation. On the other hand, off-target distribution of photosensitizers in the neibouring tissues might also caused unknown potentil toxicity, especially for those metal based photosensitizers. The prolonged retention time of the gel could maximize the therapeutic effect by maintaining and minimize the potential off-target toxicity. Biological degradation and biliverdin retention in an orthotopic glioma model should be further studied to provide more information to make the treatment plans, including NIR irradiation frequency and duration time.

We further verified the photothermal stability and retention of BVSF hydrogel in vivo (Figure 4F-G). Due to the photothermal conversion ability of biliverdin, both pre-BVSF solution and BVSF hydrogel resulted in significantly elevated surface temperature in the tumor-bearing mice under NIR irradiation. After 24 h, the surface temperature of tumors in the BVSF hydrogel group increased from 37.5 ± 1.3 °C to 52.7 ± 2.1 °C after 5 min NIR irradiation (Figure 4G), indicating the excellent photothermal stability and retention of BVSF hydrogel in vivo. This result was consistent with repeated photothermal cycle results (Figure 2C) and in vivo retention results (Figure 4C-E). However, the temperature in the pre-BVSF group only experienced a slight elevation, and even failed to reach 42~45 °C range, which is considered as the minimum temperature required for PTT. The compromised photothermal properties of pre-BVSF hydrogel could be explained by the fast clearance and resultant low intratumoral biliverdin concentration after 24 h. Therefore, BVSF hydrogel showed a good in vivo photothermal stability, which allowed repeated NIR irradiation after one dose. Repeated intracranial injections were highly risky for bacterial infections and against the following recovery. Thus, repeated PTT treatments upon one injection could be beneficial for antiglioma treatment. To sum up, the silk fibroin could form semi-solid hydrogel and prolong the retention time of biliverdin in the tumor (locally), which made it possible to exert photothermal tumor ablation by NIR irradiation-induced elevated local temperature.

### BVSF hydrogel-mediated PTT inhibiting the tumor growth

**Figure 5:**
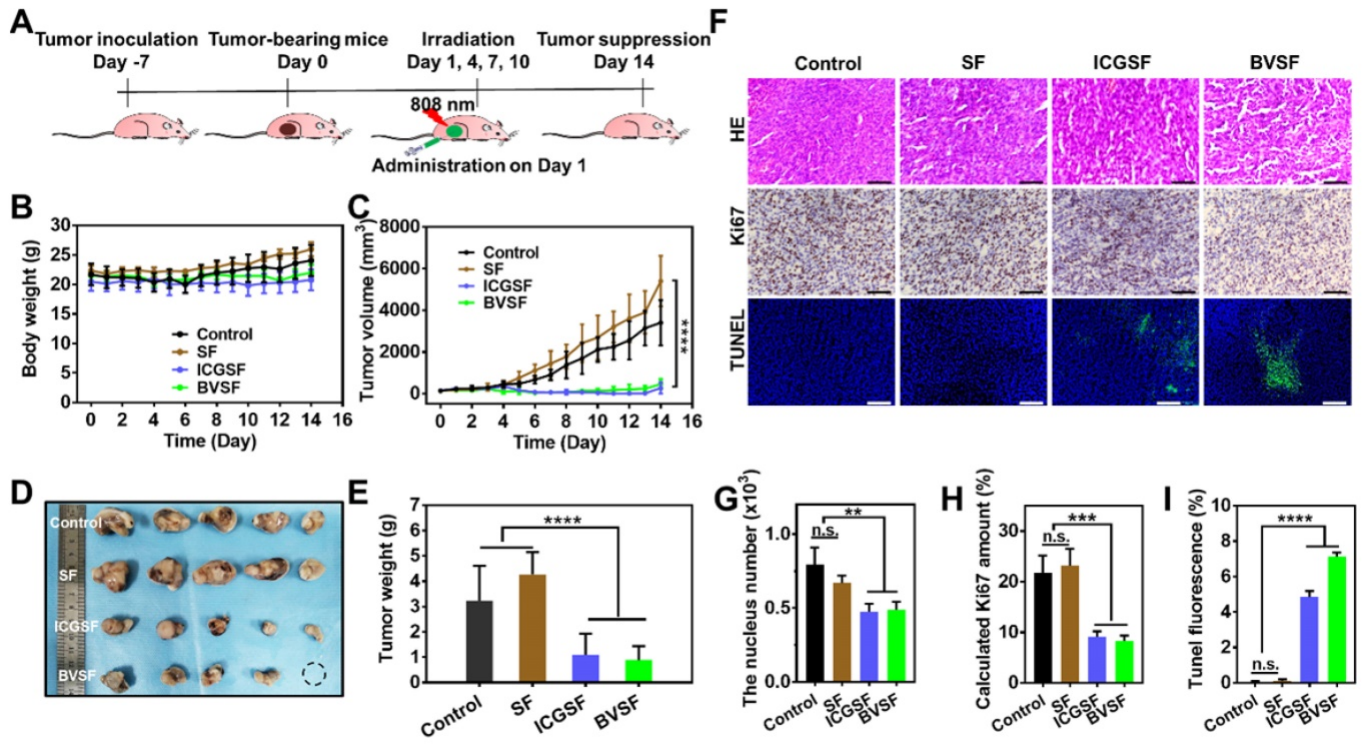
In vivo antitumor efficacy of BVSF hydrogel mediated photothermal therapy. A) The schematic diagram of in vivo antitumor study. B) Bodyweight and C) tumor growth curves following treatment with saline, SF, ICGSF, and BVSF with irradiation. D) excised tumor image and E) tumor weight at day 14 after various treatments. F) Histological analysis of the tumor tissues receiving different therapies. Scale bar =100 µm. G) Nucleus, H) Ki-67, and I) TUNEL levels were semi-quantified by using Image J software. ** represents P<0.01, *** represents P<0.001, **** represents P<0.0001.

The excellent photothermal conversion ability and prolonged tumor retention of BVSF hydrogel made it possible to be used for photothermal anticancer therapy. We then examined the anticancer effects of BVSF hydrogel in vivo (Figure 5). ICG has been widely used for photothermal therapy against cancer, and displayed effective photothermal transformation and ideal treatment efficacy. Therefore, to verify the anticancer effect of BVSF by PTT, ICG loaded SF hydrogel (ICGSF) was acted as a positive control. The nude mice were inoculated with 1×10^6^ C6 cells in the right flank. After local injection of different treatments, including saline, SF, ICGSF, and BVSF, the tumor-bearing mice received NIR irradiation with an 808 nm laser at 2 W/cm^2^ for 5 min on day 1, 4, 7, 10 (Figure 5A). The mice showed no abnormal behaviors and noticeable weight loss (Figure 5B) during the experimental period, indicating good tolerance and minimum side effects in receivers. The tumor growth in both BVSF and ICGSF was significantly inhibited during the 14 days (Figure 5C and S5), which was consistent with the tumor photograph and tumor weight results (Figure 5D-E). It was suggested that BVSF hydrogel could achieve effective inhibitory effects on tumor growth, which was comparable to the well-acknowledged ICG hydrogel. The SF itself could accelerate tumor growth to some extent, resulted in larger tumors as compared to those in the control group. This pro-growth effect of silk fibroin might be attributed to its active role in promoting cell proliferation and migration 58.

H&E staining, Ki67 staining, and TUNEL staining were conducted to perform histological analysis (Figure 5F-I). As shown in Figure 5F-G, no apoptosis was observed in the control group, whereas treatments with BVSF and ICGSF resulted in significantly reduced nucleic numbers. Immunohistochemical Ki67 staining of the tumor sections was used to evaluate the tumor cell proliferation (Figure 5F and 5H). Visually and statistically, tumor cells in the BVSF and ICGSF groups exhibited decreased Ki67 expression, indicating markedly reduced tumor cell proliferation after treatments. TUNEL staining identified that the highest portion of tumor cells with apoptotic green staining was observed in the BVSF group (Figure 5F and 5I), indicating the excellent in vivo antitumor efficiency of BVSF hydrogel-mediated PTT. These results further confirmed the BVSF hydrogel-mediated photothermal tumor ablation. Additionally, H&E staining was also conducted to study the biosafety of BVSF hydrogel. As shown in Figure S6, no visible damage was observed in the major organs that collected from the C6-bearing mice, demonstrating the excellent biosafety of the all-natural derived BVSF hydrogel.

### BVSF hydrogel accelerating wound healing in vivo

The wound healing process was often neglected during photothermal based cancer treatment. However, NIR irradiation might damage the adjacent tissue due to the leakage of the photoabsorbers, and the prolonged retention of NIR absorbing materials might hinder the local wound healing process. Moreover, the unhealed wound with continuous inflammation clearly increases difficulties for tissue regeneration. A bifunctional hydrogel that holds both photothermal properties and potent pro-healing ability provides a viable option to resolve this issue 18. Therefore, we designed a bioinspired green BVSF hydrogel for PTT. The hydrogel matrix we chose was silk fibroin, which holds the intrinsic pharmacological properties, especially facilitating cell proliferation, and has been widely used in tissue engineering and wound healing 21, 58-60. The primary photosensitizer, biliverdin, is a bioproduct and holds potent anti-inflammatory effects, which is of the practical importance for the wound healing process. We hypothesized that such an all-natural hydrogel system could be beneficial for the wound healing process after the PTT or even directly applied in wound healing.

**Figure 6:**
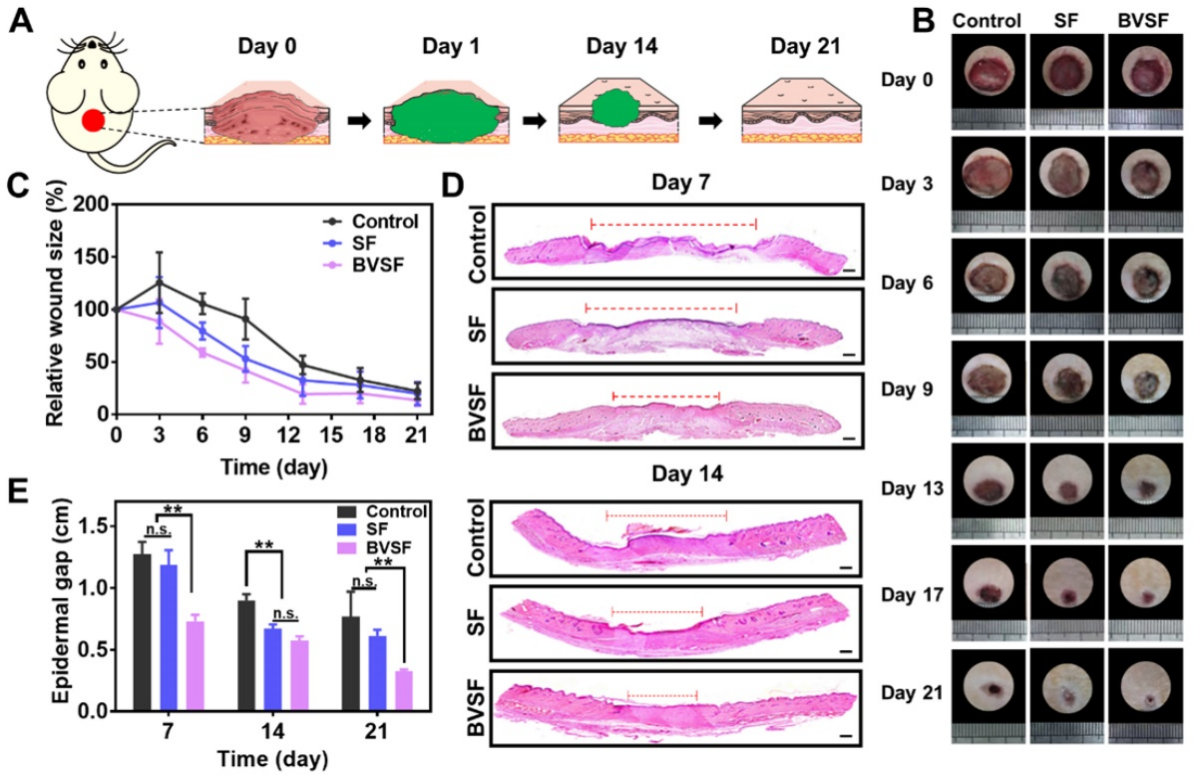
The biliverdin/silk fibroin (BVSF) hydrogel promoted the wound healing process in full-thickness skin defect rats. A) The schematic diagram of wound healing with BVSF hydrogel. B) The wound images and C) wound size change. D) H&E staining of the wounds (the wound margin was marked by the dashed line), Scale bar = 200 µm and E) the epidermal gap. ** represents P<0.01.

The performance of the BVSF hydrogel on wound healing was investigated by an in vivo model (Figure 6). The hydrogel dressing was attached to the wound bed during the experimental period (Figure 6A). The wound contraction in BVSF hydrogel group had an advantage over the control and SF group. On day 3, 6, 9, and 13, the BVSF hydrogel treated wound showed the smallest area among all groups (Figure 6B&C). Importantly, on day 6, BVSF hydrogel had an approximate 50% wound closure, which is about 20% better than the SF group and 40% better than the control group. Furthermore, it was observed that wounds in the SF group showed a faster closure rate during the healing process than the control group (Figure 6B-C), which was attributed to the pro-healing properties of silk fibroin matrix. Silk fibroin based wound dressing, including film, hydrogel, and electrospun fibrous, also showed pro-healing effects in many other published papers 21, 58-60. The accelerated wound closure in BVSF group indicated that biliverdin also played a critical role in wound healing process. To confirm the tissue repair of BVSF hydrogel on mice with full-thickness defect, H&E staining was performed, and results were displayed in Figure 6D-E and Figure S7A. Compared to both the control group and SF group, longer epithelial tongues and shorter epithelial gaps (red line) were shown in the BVSF group at day 7 and 14 (Figure 6D-E), and larger granulation tissues were observed at day 14 (Figure 6D) and 21 (Figure S7A). Additionally, the peripheral region in the BVSF group was getting well-integrated into the surrounding native skin tissue (Figure 6D and Figure S7A) in the BVSF group. The faster recovery of appendages such as hair follicles and sebaceous glands could also be observed in the H&E staining from the BVSF group. These data demonstrated that BVSF hydrogel could accelerate the wound healing in vivo, while both components, silk firoin, and biliverdin, contributed.

**Figure 7:**
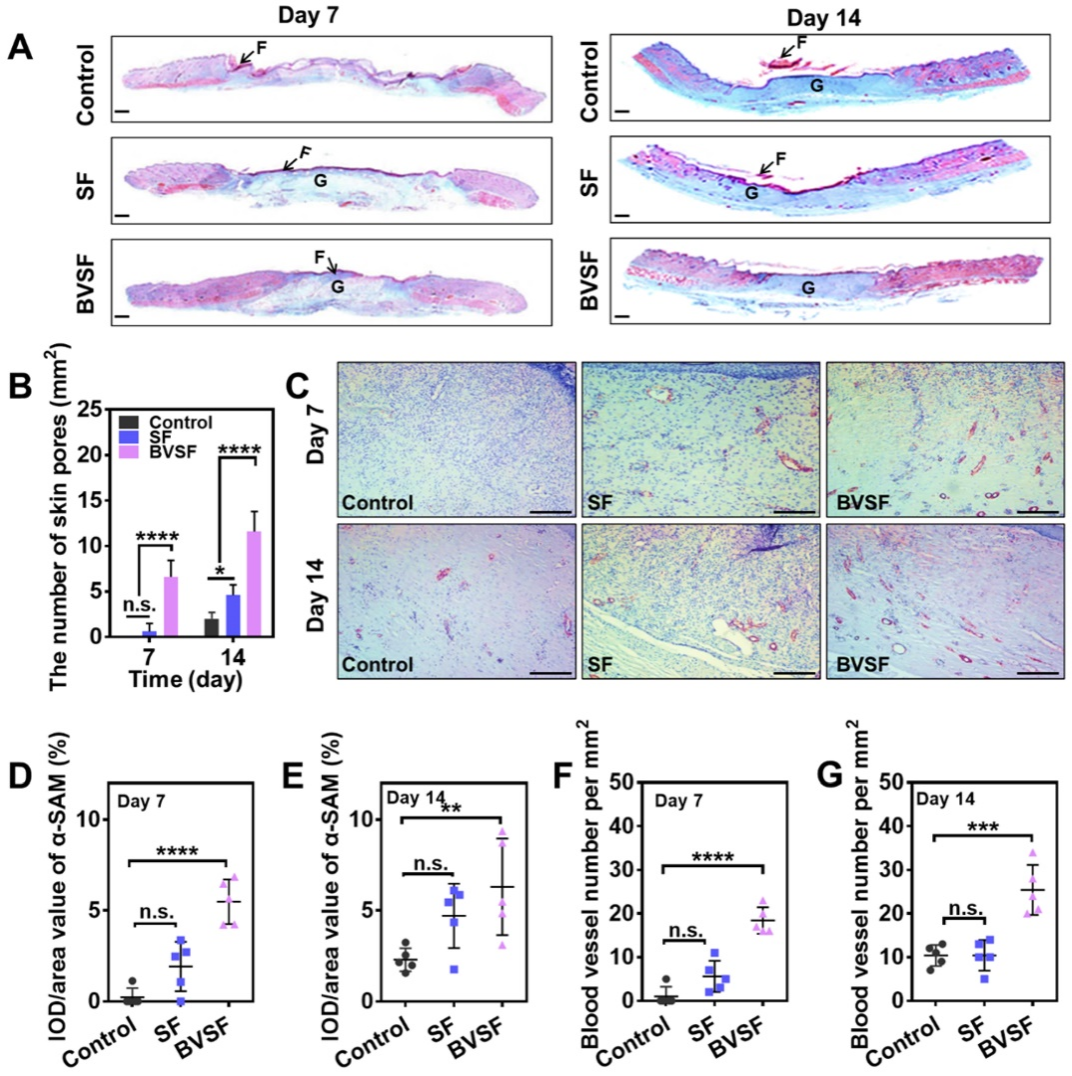
The biliverdin/silk fibroin (BVSF) hydrogel promoted the formation of myofibroblasts and new vessels. A) Masson Trichrome staining (scale bar = 200 µm) and B) the quantification of hair follicles of the skin tissue collected at day 7 and day 14. “F” indicates fibrin, and “G” indicates granular tissue. C) α-SAM immunostaining (scale bar = 100 µm) of the skin tissue collected at day 7 and day 14. The quantification of α-SAM on D) day 7 and E) day 14. The quantified analysis of blood vesicles numbers of the collected skin tissue on F) day 7 and G) day 14. ** represents P<0.01, *** represents P<0.001, **** represents P<0.0001.

Wound healing is a complex and well-programmed process, which needs a joint operation of various cell types, including macrophage, fibroblasts, keratinocytes, and endothelial cells 18. The infiltration and migration of these cells would lead to a timely inflammatory response, subsequent tissue repair, and regeneration. Collagen, a natural structural protein, participate in all stages of the healing process by its role in cellular migration and new tissue development. The replacement of the provisional extracellular matrix with a collagenous matrix has been considered a key factor for wound remodeling 61. When the skin is injured, collagen becomes the common denominator in the body's healing response. Therefore, collagen deposition was also evaluated by Masson trichrome staining (Figure 7A and Figure S7A). Masson trichrome staining revealed that denser collagen deposition and a better collagen array, approaching that of healthy skin, were found in the BVSF hydrogel group. Approximately over 80% of the wound area was collagen positive (blue color), which was similar to the collagen content in the native on day 14 (Figure S7B-D). Even though the SF group also showed increased collagen content, the effect was weaker than BVSF hydrogel. The elevated collagen content in BVSF treated wounds could be attributed to the introduction of biliverdin into the silk fibroin hydrogel system to a large extent. It has been reported that biliverdin possesses the antioxidative and anti-inflammatory properties, which might contribute to the biological process of accelerated wound healing by upregulating collagen production 35. Additionally, wounds treated with BVSF hydrogel also showed more cutaneous appendage. The statistic number of skin pores (Figure 7B and Figure S7E) also indicated a significant increasing trend during the healing process, while the highest increase rate was found in the BVSF group. In contrast, the control group exhibited limited collagen deposition and appendage regeneration at 14 days of treatment.

In general, myofibroblasts activated fibrogenic cells, were considered as one primary mediator of wound closure. Alpha-smooth muscle actin (α-SMA) was usually used as a marker for myofibroblasts. Immunohistochemistry for α-SMA (Figure 7C) revealed the expression of α-SMA after treatments. The statistical results from α-SMA staining showed that BVSF hydrogel treatment significantly increased the α-SMA expression compared to the control (Figure 7D-E). New blood vessels are also essential to wound healing by delivering oxygen and nutrients to the injured tissue microenvironment. As shown in Figure 7C, there were more blood vascular lumens observed in the BVSF group in comparison with control and SF groups, indicating denser neovascularization and a more mature capillary structure after BVSF hydrogel treatment. The number of blood vessels and tissue area occupied by vascular lumens at day 7 (Figure 7F) and day 14 (Figure 7G) was also quantified. The density of blood vessels was three times higher in the BVSF group as compared to that in the control group on day 14. As the H&E staining results, SF hydrogel treated wounds showed the basic structure of the epithelium layer and dermis layer with a few hair follicles and glands. However, the connective tissue arrangement was still not as dense as that in the BVSF hydrogel treated wounds. Furthermore, the blood vessel number in the BVSF group was almost twice as that in the SF group. Our data indicated that SF exerted some pro-healing effects on the attached wounds, which might result from its pharmacological activities. Besides that, the silk fibroin hydrogel provided excellent hemostasis, tissue repair, and a robust physical barrier to accelerate wound closure 45. The BVSF hydrogel exerted much stronger pro-healing effects as compared to the SF hydrogel, indicating that the addition of biliverdin also contributed to the therapeutic benefits of the BVSF system.

Collectively, BVSF hydrogel treated wounds underwent a faster and better wound healing via a series of physiological events, e.g., tissue repair, re-epithelization, and extracellular matrix remodeling. These data indicated that BVSF hydrogel itself could act as a wound dressing for tissue repair application, which could accelerate the wound healing during the prolonged retention in the body after the PTT.

### Anti-inflammatory effects of BVSF hydrogel

**Figure 8:**
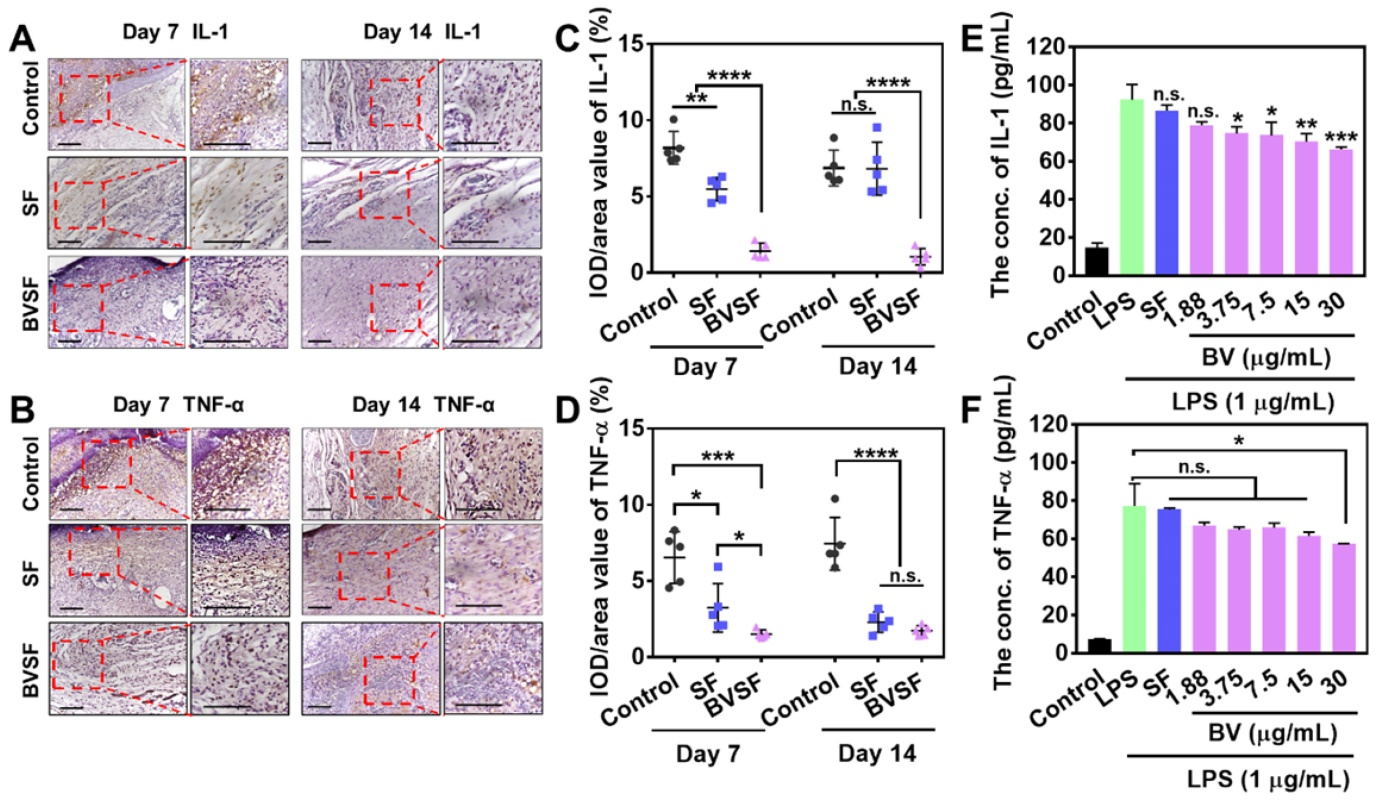
The biliverdin/silk fibroin (BVSF) hydrogel reduced the expression of pro-inflammatory cytokines. A) The IL-1 and B) TNF-α immunostaining of skin tissue collected on day 7 and day 14 (scale bar = 100 µm). Quantification of C) IL-1 and D) TNF-α on day 7 and day 14 of the collected skin tissue. The determination of E) IL-1 and F) TNF-α levels in the LPS stimulated Raw 264.7 cells with different treatments. * represents P<0.05, ** represents P<0.01, *** represents P<0.001, and **** represents P<0.0001.

Many studies have demonstrated that inflammatory cytokines are highly involved in the regulation of multiple cellular activities during the initiation and cessation of wound healing 62. The in vivo study and pathological experiments indicated that BVSF hydrogel could accelerate the healing process. The results indicated that silk fibroin itself could facilitate tissue remodeling and revascularization to some extent, which was consistent with other reports 58. Biliverdin, as one main component in the BVSF hydrogel system, also contributed to the wound repair. Our previous research indicated that bilirubin possesses a power anti-inflammatory property, which showed significant cytoprotective effects against oxidative and inflammatory damage in multiple cell lines 40, 63-65. Biliverdin, as a precursor substance of bilirubin, has also been demonstrated as an important cytoprotective and anti-inflammatory molecule 34, 35. Then, we further used immunostaining to evaluate the level of pro-inflammatory cytokines, including IL-1 and TNF-α, which play an essential role in wound repair (Figure 8A-B). The statistical analysis showed that the expression of IL-1 and TNF-α were significantly down-regulated in the BVSF treated wounds on both day 7 and day 14 (Figure 8C-D), indicating that BVSF hydrogel could exert anti-inflammatory effects on the attached wounds.

Macrophages play key roles in the wound-healing process, especially boosting host defenses, promoting and resolving inflammation 66, 67. Whereas, excessive inflammatory response mediated by macrophages restricts the wound healing process 68. Therefore, we further evaluated the effect of the components from BVSF hydrogel on the expression of inflammatory cytokines using LPS-induced RAW 264.7 cells (Figure 8E-F). The levels of both IL-1 (Figure 8E) and TNF-α (Figure 8F) were significantly increased after LPS stimulation. The addition of biliverdin at a concentration of 30 µg/mL significantly decreased the levels of both two inflammatory cytokines. Additionally, the anti-inflammatory effect of biliverdin showed a concentration dependency. Also, the addition of silk fibroin had no apparent effect on the expression of IL-1 and TNF-α in the LPS activated RAW 264.7 cells. Therefore, the BVSF hydrogel could inhibit the expression of IL-1 and TNF-α during the wound healing process, which mainly attributed to the introduction of biliverdin into the hydrogel system. Collectively, BVSF hydrogel could accelerate the wound healing process regarding wound closure rate, collagen deposition, epidermis, hair follicles, and neovascularization by inhibiting the inflammation, promoting the tissue remodeling, and facilitating vascularization and cell proliferation. What should be mentioned is that some studies reported that wound healing would support survival of the tumor, which is not beneficial for the tumor treatment. In our study, the tumor ablation was triggered by NIR irradiation mediated PTT (Figure 3&5). As shown in the tumor growth curves (Figure 5A), the promoting wound healing property could be neglected during the treatments. When applied in an orthotopic model or future clinical use, BVSF was firstly used to remove residual cancer cells by PTT after surgical resection. After that, the remained BVSF hydrogel would promote the healing of the large wound resulted from surgical resection and PTT. This is the rationale for combining cancer treatment and wound healing.

In this study, we successfully developed a bioinspired green BVSF hydrogel by incorporating biliverdin into the silk fibroin matrix. Unlike other photothermal formulations, our hydrogel was manufactured with two natural derivatives: (i) biliverdin, a common bioproduct that possesses anti-inflammatory property, and (ii) silk fibroin, a natural protein that has been explored for various medical applications with excellent biocompatibility and biodegradability. In human body, biliverdin interacted with myoglobin and iron and formed as hemoglobin. Therefore, we used a protein-based biomaterial, silk fibroin, to formulate biliverdin and increase the photothermal conversion efficiency. To the best of our knowledge, this is the first work that employed biliverdin as a NIR absorbing photothermal agent without metal ion for PTT. Fathi et al. used nanoprecipitation method to prepare bifunctional amine linker-crosslinked biliverdin nanoparticle, which showed strong photoacoustic signal and complete metabolic digestion 37. Whereas they did not investigate the photothermal effect of biliverdin nanoparticles. Xing at al. utilized biliverdin, metal-binding short peptides, and Mn^2+^ to construct the supramolecular multicomponent biliverdin nanoagents, which showed intense NIR absorption, long-term photostability and colloidal stability, and high photothermal conversion efficiency 4. Lee et al. developed a cisplatin-chelated bilirubin-based nanoparticle for combined photoacoustic imaging and photothermal therapy of cancers, whereas they used bilirubin, not biliverdin 69. Even though metal ion-contained biliverdin/bilirubin formulations have achieved ideal photothermal therapy, the addition of metal ions raises the health concerns for the healing process, especially during the post-treatment prolonged retention period. In our study, the biliverdin not only acted as a localized photosensitizer for the thermal ablation of residual cancer cells but also played an active role in promoting the healing regenerative processes. Additionally, the self-bioactive silk fibroin also could facilitate wound healing. Our results indicated that BVSF hydrogel described here was a promising biocompatible system that can be used for effective photothermal cancer therapy and following local wound healing along with tissue repair. As for the future clinical application, BVSF could be placed at the tumor site after glioma excision by surgery. Following that, NIR irradiation could be used to induce PTT to ablate residual cancer cells, and this noninvasive way would be feasible and advantageous to treat unresectable glioma cell and avoid recurrence. Enough irradiation time and frequency should be designed in the workflow to remove all the residual cancer cells, and then the therapeutic regimen could be stopped. It was expected that PTT would be performed within 1 week after surgery every day or every two days considering the patient condition, adaptability, and compliance. The remained BVSF hydrogel could facilitate the healing process of the large wound resulting from the surgical resection and PTT of glioma.

## Conclusions

In summary, we have successfully prepared an all-natural green BVSF hydrogel by the integration of biliverdin into the silk fibroin matrix for anti-glioma PTT and subsequent tissue regeneration. With the presence of biliverdin, BVSF hydrogel could quickly raise the temperature beyond 45 °C under NIR irradiation, enabling killing effect on cancer cells in vitro and inhibitory effect on tumor growth in vivo. Meanwhile, the BVSF hydrogel could stimulate cell proliferation, migration, and adhesion along with possessing anti-inflammatory properties, and significantly accelerate wound repair and regeneration. Therefore, the bioinspired BVSF hydrogel system offers a viable alternative for PTT and post-treatment care, which address the current photothermal induced wound healing issues.

## Supplementary Material

Supplementary figures.
