# Supplementary material for: Bioinspired biliverdin/silk fibroin hydrogel for antiglioma photothermal therapy and wound healing
Source: Theranostics. 2020 Sep 23;10(25):11719–36. doi: 10.7150/thno.47682 (PMC7545989; doi:10.7150/thno.47682)
Supplement: Supplementary file 1 — Supplementary figures. [file thnov10p11719s1.pdf]

Supplementary Materials

Bioinspired biliverdin/silk fibroin hydrogel for antiglioma photothermal therapy and wound healing

Qing Yao [1,2,#], Qing-Hua Lan[2,#], Xinyu Jiang [3], Chu-Chu Du [2], Yuan-Yuan Zhai [2], Xiaohan Shen [4], He-Lin Xu [2], Jian Xiao [2], Longfa Kou[3,], Ying-Zheng Zhao [1,2*]*

[1] Department of Ultrasonography, The First Affiliated Hospital of Wenzhou Medical University, Wenzhou, Zhejiang, China

[2] School of Pharmaceutical Sciences, Wenzhou Medical University, Wenzhou 325035, China

[3] Department of Pharmacy, The Second Affiliated Hospital and Yuying Children's Hospital of Wenzhou Medical University, Wenzhou 325035, China

[4] Department of Oncology, Affiliated Cixi Hospital, Wenzhou Medical University, Cixi, 315300, China.

[#]These authors contributed equally to this work.

*Corresponding authors:

Longfa Kou, Wenzhou Medical University, University Town, Wenzhou 325035, China; Email: klfpharm@163.com;

Ying-Zheng Zhao, Wenzhou Medical University, University Town, Wenzhou 325035, China; Email: zyzpharm@163.com

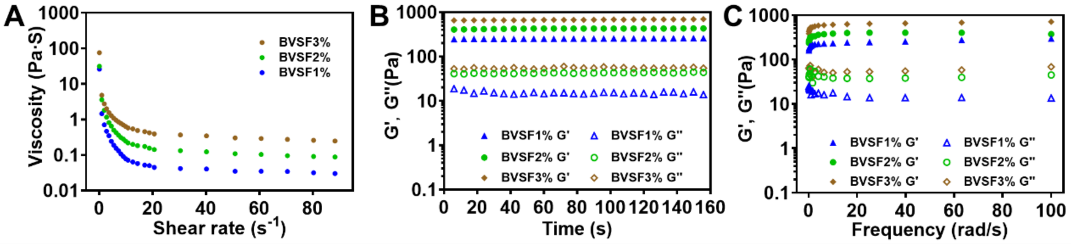

Figure S1. The rheological properties of BVSF hydrogels with 1%, 2%, and 3% concentration. (A) The viscosity of BVSF hydrogels with the shear rate from 0.1 to 100 s^{-1}. (B) The rheological behavior of BVSF hydrogels swept by time. (C) The rheological behavior of BVSF hydrogels swept by frequency.

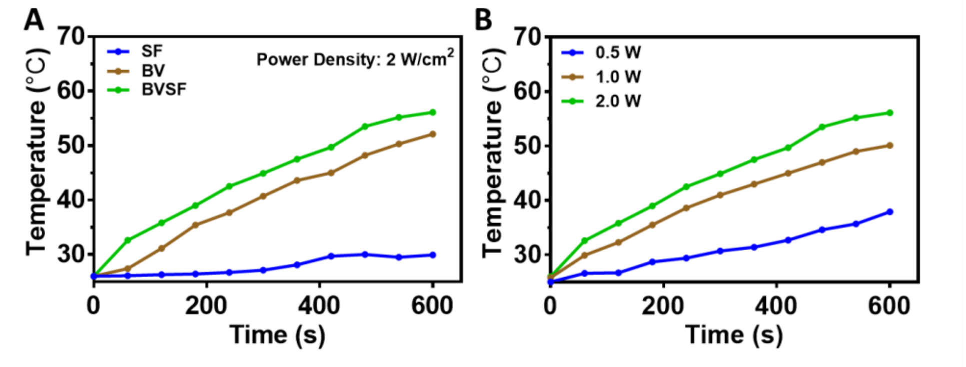

Figure S2. The photothermal properties of BVSF hydrogel. (A) The photothermal heating curves of silk fibroin solution, biliverdin solution and BVSF hydrogel by NIR irradiation (808 nm, 2 W/cm^2 laser); (B) The photothermal heating curves of BVSF hydrogel by NIR irradiation with different power.

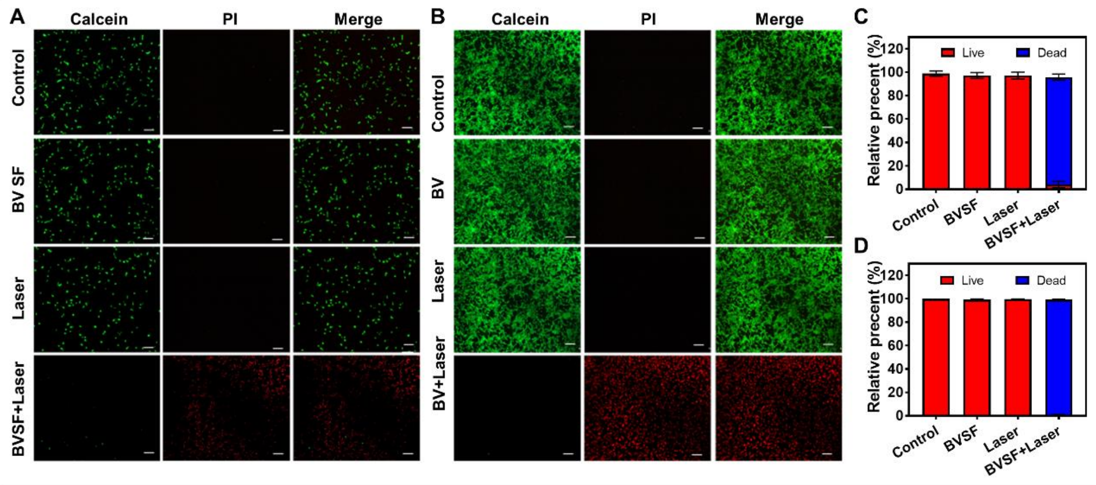

Figure S3. The anticancer effect of BVSF hydrogel on GL261 and U87 cells with NIR irradiation (808 nm, 2 W/cm^2). The live/dead staining images on (A) GL261 and (B) U87 cells (green: live cells; red: dead cells). Scale bar = 100 µm. The calculated live/dead ratio of (C) GL261 and (D) U87 cells.

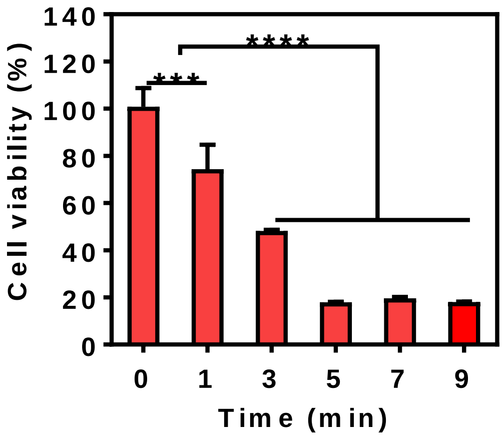

Figure S4. Relative cell viability of C6 cells after BVSF treatment with different NIR irradiation time.

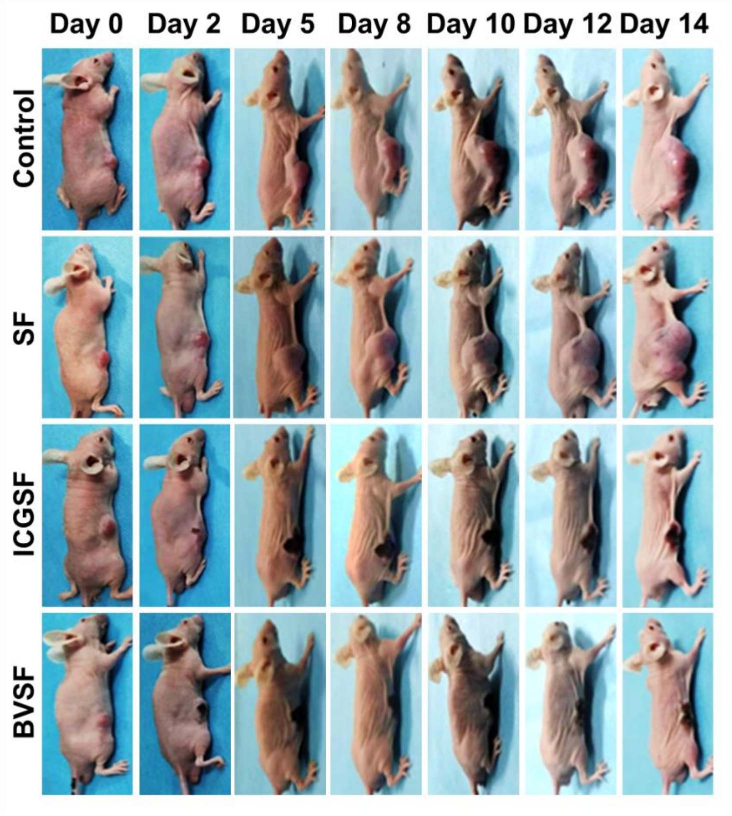

Figure S5. The photographs of C6-bearing mice receiving different antitumor therapies.

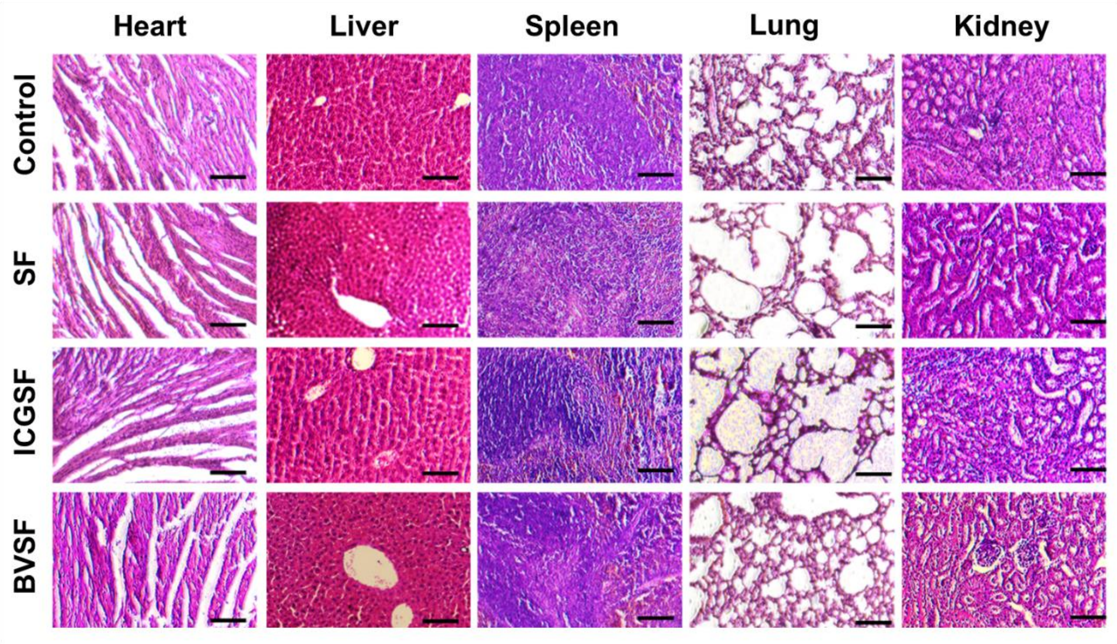

Figure S6. H&E staining of the major tissues (heart, liver, spleen, lung, and kidney) collected from C6-bearing mice after different therapies (scale bar = 100 μm).

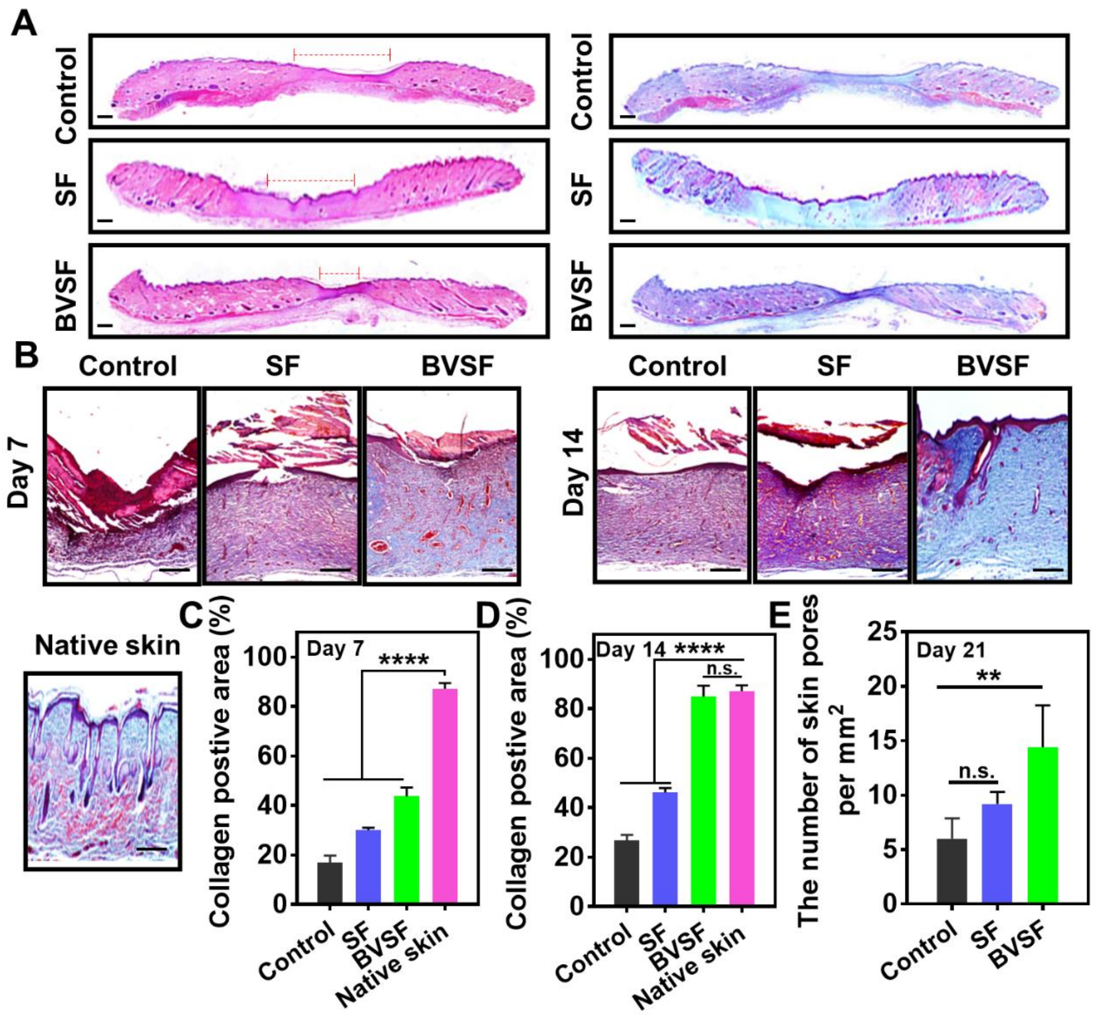

Figure S7. Historical staining of the healing wounds in full-thickness skin defect rats. A) H&E staining and Masson trichrome staining of the healing wounds (the wound margin was marked by dashed line) at day 21. Scale bar: 200 µm. B) Masson trichrome staining of the healing wounds at day 7 and day 14. The blue color indicates collagen staining. Collagen-positive areas were normalized to the total areas of the skin tissue. Scale bar = 100 µm. C) Quantification of Collagen-positive in areas at day 7; D) Quantification of Collagen-positive in areas at day 14; E) Quantification of hair follicles in number at day 21.
